# Cytokine Networks and Clinical Heterogeneity in Sjögren’s Disease: From Glandular Inflammation to Therapeutic Stratification

**DOI:** 10.3390/ijms27156638

**Published:** 2026-07-25

**Authors:** Eui-Jong Kwon, Bong-Woo Lee, Ji Hyeon Ju

**Affiliations:** 1 Division of Rheumatology, Department of Internal Medicine, Bucheon St. Mary’s Hospital, College of Medicine, The Catholic University of Korea, Bucheon 14647, Republic of Korea; ejkwon@catholic.ac.kr; 2 Division of Rheumatology, Department of Internal Medicine, Seoul St. Mary’s Hospital, College of Medicine, The Catholic University of Korea, Seoul 06591, Republic of Korea

**Keywords:** Sjögren’s disease, Sjögren’s syndrome, cytokines, interferon, BAFF, B cell activation, salivary gland epithelial cells

## Abstract

Sjögren’s disease (SjD) is a chronic autoimmune disease characterized by lymphocytic infiltration and dysfunction of the exocrine glands, with manifestations extending beyond glandular sicca symptoms to multiple extraglandular systems. Although the pathogenesis of SjD remains incompletely understood, growing evidence indicates that a complex cytokine network involving both innate and adaptive immune pathways plays a central role in disease development. This narrative review summarizes recent updates on cytokine signaling in SjD across three clinically relevant domains. In glandular inflammation, activation of salivary gland epithelial cells through Toll-like receptor pathways triggers type I interferon (IFN) signaling via plasmacytoid dendritic cells, while IFN-γ, Th17-related cytokines (IL-6, IL-17, IL-22), BAFF/APRIL, and chemokines (CXCL10, CXCL12, CXCL13) collectively sustain local inflammation and ectopic lymphoid organization. The BAFF/APRIL axis, a systemic type I IFN signature, and IL-21–follicular helper T cell–B cell interactions primarily drive systemic immune activation, which together underlie autoantibody production, hypergammaglobulinemia, and a lymphoma-prone phenotype. In contrast, constitutional symptoms such as fatigue, pain, and dryness frequently dissociate from classical inflammatory activity and are better explained by neuroimmune–metabolic mechanisms, including the IFN-γ–IDO–kynurenine pathway and symptom-associated proteomic signatures. Collectively, these findings underscore the heterogeneous nature of SjD, in which glandular inflammation, systemic immune activation, and constitutional symptoms are driven by distinct yet partially overlapping cytokine pathways. Recognizing this heterogeneity has direct implications for cytokine-targeted therapy, suggesting that future trials should stratify patients by disease phenotype (IFN-high, B cell-dominant, and symptom-dominant) rather than treating SjD as a uniform population.

## 1. Introduction

Sjögren’s syndrome (SjD) is a chronic autoimmune disease characterized by lymphocytic infiltration and subsequent dysfunction of the exocrine glands, particularly the lacrimal and salivary glands [1]. Beyond its cardinal glandular manifestations, including keratoconjunctivitis sicca and xerostomia, SjD can involve multiple extraglandular systems, including the musculoskeletal, cutaneous, peripheral nervous, hematologic, and pulmonary systems [2].

Classically, SjD has been categorized according to the presence or absence of other systemic autoimmune rheumatic diseases (SARDs) [1,2]. Primary SjD (pSjD) occurs without SARDs, whereas secondary SjD occurs in the presence of SARDs such as rheumatoid arthritis, systemic lupus erythematosus, and systemic sclerosis [2,3]. pSjD and secondary SjD have distinctive features in their clinical course and treatment targets [2,3]. Recently, the use of the term “secondary” has been considered potentially confusing in the context of SjD, and “associated SjD” is increasingly favored to describe this disease entity [3]. Additionally, the term “Sjögren’s disease” has been proposed as a replacement for “Sjögren’s syndrome” to better reflect its nature as a systemic autoimmune disease [3].

The pathogenesis of SjD remains incompletely understood, and it remains debated whether its clinical and molecular heterogeneity justifies further stratification into distinct disease subgroups [2,4]. Genetic factors such as human leukocyte antigen DQB1 (HLA-DQB1), interferon regulatory factor 5 (IRF5), signal transducer and activator of transcription 4 (STAT4), B lymphocyte kinase (BLK), interleukin-12 subunit alpha (IL12A), TNFAIP3-interacting protein 1 (TNIP1), and C-X-C chemokine receptor type 5 (CXCR5); epigenetic events including DNA hypomethylation, histone acetylation, and microRNA expression; and hormonal factors including female sex hormones may contribute to disease susceptibility [4,5]. Environmental factors such as viral infection (e.g., Epstein–Barr virus, cytomegalovirus, and Coxsackievirus), solvents, and inorganic chemicals may also contribute to disease development [6].

In SS, salivary gland epithelial cells (SGECs) function both as targets of immune-mediated tissue injury and as active initiators of local immune activation [7,8]. Upon activation through Toll-like receptor (TLR) signaling pathways, SGECs release pro-inflammatory cytokines and damage-associated molecular patterns (DAMPs), thereby promoting the recruitment of innate immune cells [8,9,10]. These recruited innate immune cells then amplify cytokine signaling, thereby driving adaptive immune activation and subsequent tissue damage [7,8]. These complex cytokine networks form a central framework of SjD pathophysiology [7,8].

Currently, the treatment of SjD focuses on relief of symptoms, screening for complications such as lymphoma or interstitial lung disease, and control of underlying SARDs in cases of secondary SjD [11]. Although there is no approved disease-modifying therapy for SjD, several therapeutic candidates have been developed to prevent damage to the exocrine glands and improve patients’ quality of life, with some already entering clinical trials [12,13,14,15,16]. However, the pathogenesis of SjD remains incompletely understood, which continues to limit the development of effective treatments [7,11].

Therefore, this narrative review focuses on recent updates regarding cytokine signaling and related molecules involved in SjD. Cytokine networks in SjD can be discussed in relation to three clinically relevant domains: glandular inflammation, extraglandular systemic activity, and constitutional symptoms such as fatigue, pain, and dryness [7,8,9,17,18,19,20,21,22].

Before turning to cytokine networks within specific clinical domains, it helps to distinguish three functional components of the network: upstream initiating signals, downstream effector or amplification pathways, and feedback mechanisms [7,8,23,24]. Upstream signals comprise epithelial TLR activation, DAMP release, immune-complex stimulation, and pDC-driven type I IFN production [7,8,9,10,24,25]. Downstream effector pathways span IFN-JAK/STAT signaling, IFN-γ-Th1/NK-cell responses, IL-6-Th17/IL-17/IL-22-related inflammation, BAFF/APRIL-driven B cell activation, and CXCL chemokine axes [19,21,23,24,25,26,27,28,29,30,31]. Feedback mechanisms encompass epithelial injury, autoantigen exposure, B cell activation, immune-complex formation, and ectopic lymphoid organization [7,8,10,24,27,30,32,33,34].

**Figure 1:**
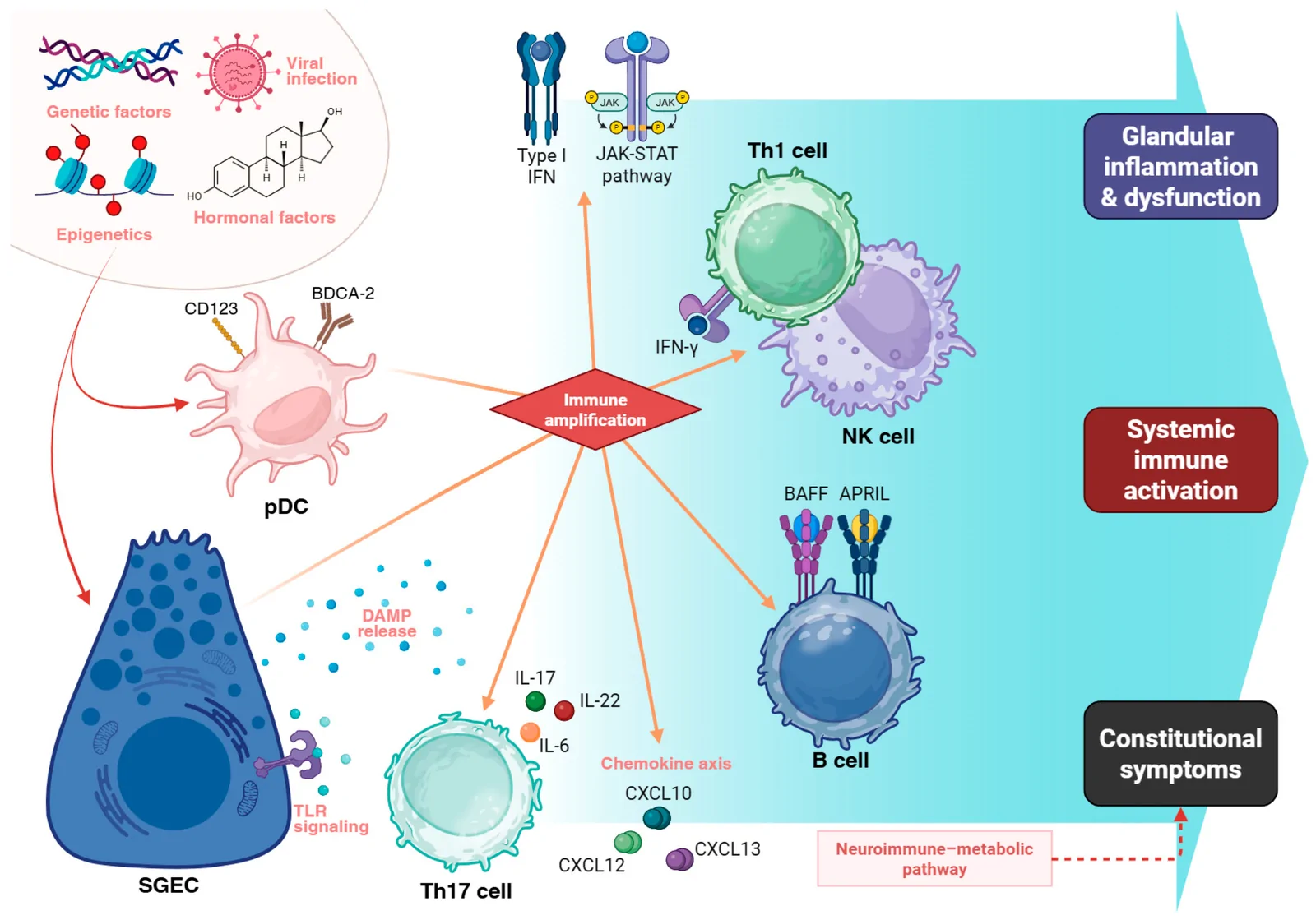
Cytokine networks across clinical domains in Sjögren’s disease. Genetic, epigenetic, hormonal, and viral factors contribute to salivary gland epithelial cell (SGEC) activation. Activated SGECs release damage-associated molecular patterns (DAMPs) and pro-inflammatory mediators through Toll-like receptor (TLR)-related pathways. Plasmacytoid dendritic cells (pDCs) promote type I interferon (IFN) production and JAK–STAT pathway activation. In parallel, IFN-γ-producing Th1 and natural killer (NK) cells, IL-6/IL-17/IL-22-related Th17 responses, BAFF/APRIL-mediated B cell activation, and CXCL10/CXCL12/CXCL13 chemokine axes contribute to glandular inflammation and systemic immune activation. Constitutional symptoms may be linked partly to neuroimmune–metabolic pathways rather than classical inflammatory cytokine activity alone. The dotted arrow indicates a proposed and indirect link between immune activation and constitutional symptoms through neuroimmune–metabolic pathways, rather than a direct or fully established cytokine-mediated inflammatory pathway. Abbreviations: APRIL, a proliferation-inducing ligand; BAFF, B cell-activating factor; CXCL, C-X-C motif chemokine ligand; DAMP, damage-associated molecular pattern; IFN, interferon; IL, interleukin; JAK, Janus kinase; NK, natural killer; pDC, plasmacytoid dendritic cell; SGEC, salivary gland epithelial cell; STAT, signal transducer and activator of transcription; Th, T helper; TLR, Toll-like receptor.

Because these components interact extensively rather than following a strictly linear sequence, however, the remainder of this review is organized around clinically relevant disease domains—glandular inflammation, systemic immune activation with extraglandular manifestations, and constitutional symptoms—so that the same cytokine axis can be examined across different pathogenic contexts while still preserving the distinction between initiating signals, effector pathways, and feedback loops [7,8,9,17,18,19,20,21,22]. Figure 1 outlines how epithelial activation, immune amplification, and these clinical domains connect in SjD.

## 2. Cytokines Involved in Glandular Inflammation

Exocrine gland inflammation, particularly within the salivary and lacrimal glands, cannot be explained solely by immune cell infiltration [7]. SGECs are not only targets of immune-mediated organ damage, but also initiators and amplifiers of immune responses [8,10]. In genetically susceptible individuals, viral infections or environmental stimuli activate TLRs on SGECs, followed by the release of DAMPs and pro-inflammatory cytokines [9,10]. Furthermore, adhesion and co-stimulatory molecules such as CD40, ICAM-1, and VCAM-1 are upregulated [7,8,9,10]. This cascade establishes a local inflammatory environment, facilitating tissue infiltration by dendritic cells, T cells, and B cells [7,8]. Together, SGECs are central contributors to the development of “autoimmune epithelitis” [7].

Epithelial activation in the early phase of SjD pathogenesis triggers innate immune amplification, with type I interferon serving as a central mediator [7,22]. After apoptosis or necrosis of SGECs, nucleic acid-containing materials are released. These materials bind to autoantibodies against RNA-binding proteins and form immune complexes that stimulate TLR7/TLR9 of plasmacytoid dendritic cells (pDCs) [7,9,22]. Activated pDCs subsequently produce large amounts of interferon-α (IFN-α), thereby amplifying innate immune signaling [22,35]. IFN-α induces the expression of interferon-stimulated genes (ISGs) via the JAK-STAT pathway, which represents a key molecular signature of SjD pathophysiology [22,35].

Evidence of increased JAK-STAT pathway signaling and ISG expression has been demonstrated in both minor salivary glands and peripheral blood mononuclear cells (PBMCs) in studies based on human tissue [22,35,36]. In these human tissue-based studies, IFN signaling pathway activity also correlated with the focus score of minor salivary gland biopsy and anti-Ro antibody positivity [22,35]. Single-cell analysis revealed that this IFN-related signal reflects a cell-type-specific phenomenon, occurring in epithelial cells, infiltrating lymphocytes, antigen-presenting cells (APCs), and endothelial cells [37]. Transcriptomic and molecular subset analyses further indicate that this heterogeneity extends across patients, with IFN-related signals varying rather than uniformly present [36,38]. Collectively, these data suggest that IFN activation may define a molecular subset of SjD rather than a universal disease feature [22,35,36,38].

IFN-γ represents another signaling axis that is partly independent of type I IFN [22]. Conventional dendritic cells (DCs) and APCs produce IL-12, which induces the differentiation of CD4+ T cells into T helper 1 (Th1) cells [23,27]. Differentiated Th1 cells and natural killer (NK) cells become the major sources of IFN-γ production [23,27]. IFN-γ acts on both SGECs and infiltrating immune cells, epithelial activation, and increasing expression of major histocompatibility complex molecules and inflammatory mediators, thereby perpetuating local inflammation [27]. Indeed, IFN-γ worsens epithelial barrier dysfunction, tight junction impairment, and cytotoxic tissue injury in concert with tumor necrosis factor-α (TNF-α) [27]. CD8+ T cells and NK cells produce cytotoxic mediators, inducing SGEC apoptosis and tissue injury [7,8]. Injured epithelial cells expose autoantigens and DAMPs, and these exposed molecules re-stimulate innate immune activation, forming a vicious cycle [7,8]. These pathways support the notion that IFN-γ is not merely a co-stimulatory cytokine acting alongside type I IFN, but rather an effector cytokine bridging T cell/NK cell-mediated epithelial injury and salivary gland dysfunction [23,27,31].

Th17-Treg imbalance is thought to drive chronic inflammation of the exocrine glands in SjD [23,34]. IL-17 secreted by Th17 cells stimulates SGECs and glandular stromal cells, amplifies inflammatory mediators, and compromises the epithelial barrier [31,32,34]. Consistent with this, an experimental study found that IL-17 disrupted salivary tight junction integrity, pointing to a role downstream of epithelial dysfunction [31]. Th17 cells also co-produce IL-22, and serum IL-22 has been associated with disease activity and lymphocyte profiles in pSjD [21,34]. Normally, regulatory T cells hold Th1/Th17-skewed inflammation in check, largely through IL-10 and TGF-beta, so any decline in Treg function could allow inflammation to persist unchecked [23]. The IL-6-Th17 axis, Treg-Th17 balance, and IL-22 signaling therefore appear to act in concert to sustain glandular inflammation and drive lymphoid organization [21,23,32,34].

More recently, attention has turned to the gut microbiome as a possible modifier of this Th17-Treg balance [24,25,39]. Patients with pSjD show reduced gut microbial diversity, with notable depletion of butyrate producers such as *Roseburia* and *Coprococcus* [24,39]. One study reported an inverse correlation between Th17 cell levels and abundance of these two genera, tying microbial shifts directly to Th17/Treg imbalance [24]. Other work has linked gut dysbiosis to systemic inflammation, increased gut permeability, and changes in FOXP3 expression alongside disease activity [25,39]. Microbiota-derived metabolites may well shape mucosal immune tone, but at this stage the gut microbiome is better viewed as a contributing modulator than a core driver of SjD pathogenesis [24,25,39].

B cell-activating factor (BAFF) and a proliferation-inducing ligand (APRIL) are key cytokines that maintain B cell survival and plasma cell differentiation in glandular lesions [17,20,28]. Classically, monocytes, macrophages, and dendritic cells are recognized as major sources of BAFF production; however, SGECs of SjD patients can also produce BAFF [17,18,20,28]. Considering the notion that the type I IFN pathway induces BAFF expression, IFN activation becomes a mediator of B cell hyperactivity [18]. In salivary glandular tissue, BAFF and APRIL support the maturation and survival of B cells, maintaining plasma cell survival and local autoantibody production [20]. In this manner, BAFF and APRIL enhance glandular B cell-rich infiltration and ectopic germinal center-like structure formation [17,20,28]. These cytokines are not solely indicators of systemic B cell activation, but rather core molecules in the cytokine environment supporting the local B cell niche in the salivary glands of SjD [17,18,20].

The chemokine network drives the overt manifestation of immune cell infiltration and structural change from this cytokine-driven inflammation [19]. CXCL10 is related to a Th1-oriented response as an IFN-responsive chemokine [19]. Conversely, CXCL12/13 are connected to B cell recruitment and ectopic lymphoid structure formation [19,21]. IL-8 promotes leukocyte infiltration after TNF-α-driven epithelial activation [19]. Thus, glandular inflammation represents the final convergence of a cytokine-chemokine network, where SGEC activation, IFN signature, Th1-Th17 polarization, BAFF/APRIL-mediated B cell survival, and chemokine-driven immune cell trafficking collectively orchestrate a self-perpetuating inflammatory milieu [19,20,21].

Collectively, glandular inflammation in SjD can be conceptualized as a hierarchical yet non-linear network [7,8,19]. Upstream initiating signals include epithelial TLR activation, DAMP release, immune-complex stimulation, and pDC-derived type I IFN [9,10,22,35]. These signals converge on downstream effector and amplification pathways, including IFN–JAK/STAT signaling, IFN-γ–Th1/NK-cell responses, IL-6–Th17/IL-17/IL-22-related inflammation, BAFF/APRIL-mediated B cell activation, and CXCL chemokine-mediated immune-cell trafficking [17,18,19,20,21,22,23,27,28,31,34,35,36]. Epithelial injury, autoantigen exposure, B cell activation, immune-complex formation, and ectopic lymphoid organization subsequently establish feedback loops that perpetuate local inflammation [7,20,29]. This framework highlights that glandular disease in SjD is sustained by a network of cytokine axes rather than by any single dominant pathway.

## 3. Cytokines Associated with Systemic Immune Activation and Extraglandular Manifestations

Extraglandular disease manifestations are characterized more prominently by systemic immune activation and B cell hyperactivity than by glandular inflammation [28]. In SjD patients, the continuum of B cell activation is demonstrated by autoantibody production, hypergammaglobulinemia, circulating plasmablast and plasma cell expansion, hypocomplementemia, cryoglobulinemia, and a lymphoma-prone phenotype [28]. During this process, BAFF/APRIL serves as the key cytokine axis promoting B cell survival, maturation, and plasma cell maintenance [17,20,28]. Elevated BAFF levels, detected in serum and salivary gland tissue, are widely reported and correlated with anti-Ro and anti-La antibodies [17,20,28]. In this regard, BAFF/APRIL extends beyond local glandular mediators, functioning as cardinal cytokines that sustain systemic B cell hyperactivity and shape autoantibody-positive phenotypes [17,20,28].

A systemic type I IFN signature represents one of the principal molecular features underlying extraglandular manifestations of SjD [22,38]. IFN activation is not confined to the salivary gland, but extends systemically, as evidenced by its presence in PBMCs [22,38]. Peripheral upregulation of ISGs is reported in numerous innate immune cells, especially in monocytes, DCs, and NK cells [22]. This IFN signature contributes to systemic immune activation, autoantibody-positive phenotype, and B cell activation [22,38]. Following type I IFN amplification of glandular inflammation, it establishes a cytokine axis that sustains systemic inflammatory tone across serum and tissue [22,38]. However, it is an overinterpretation to directly correlate each extraglandular manifestation with a specific IFN pathway; rather, the IFN signature functions as a background reflection of systemic autoimmunity and molecular endotype [22,36,38].

The IL-21–follicular helper T (Tfh) cell–B cell interaction represents another crucial adaptive immune mechanism driving systemic autoimmunity, alongside the BAFF/APRIL axis [26,29]. Tfh cells secrete IL-21 and IL-4, accelerating B cell proliferation and class switching, ultimately culminating in high-affinity autoantibody production [26]. These cascades are reflected in anti-Ro positivity, hypergammaglobulinemia, and tissue infiltrate complexity of SjD [29]. Impaired balance between Tfh cells and follicular regulatory T (Tfr) cells underlies the failure to modulate germinal center reactions, perpetuating persistent autoantibody production [26,29]. IL-21 is not an independent inflammatory cytokine, but rather a mediator bridging B cell activation to humoral autoimmunity—a conceptual reframing that more precisely captures its pathogenic role in SjD [26,29].

Th17/Treg imbalance, IL-6, and IL-17 each contribute independently to systemic immune dysregulation, extending their pathogenic roles beyond glandular inflammation into extraglandular manifestations [23,32,40]. IL-6 promotes Th17 differentiation and weakens Treg stability, tipping the immune milieu toward a pro-inflammatory state [23]. IL-17, as a downstream effector of Th17-skewed immunity, further amplifies disease activity in systemic inflammatory conditions [32]. In addition, the decline of the Breg/Treg axis, which produces IL-10 and IL-35, results in the loss of suppression of Th1 and Th17 responses, perpetuating a pro-inflammatory cytokine environment [23]. In this context, extraglandular manifestations are the result of a collapsed equilibrium among diverse molecular mediators—IFN, BAFF/APRIL, IL-21, IL-6/IL-17, and other regulatory cytokines [22,32,40].

Although extraglandular manifestations are clinically heterogeneous, the cytokine pathways discussed above should not be interpreted as organ-specific mechanisms in a strict one-to-one manner; rather, pulmonary, cutaneous, neurologic, hematologic, and lymphoproliferative involvement appear to arise against a shared background of systemic immune activation [2,17,20,22,28,29]. In this regard, BAFF/APRIL, the type I IFN signature, IL-21–Tfh cell–B cell interaction, and IL-6/Th17-related inflammation are more appropriately regarded as systemic immunologic contexts that increase the likelihood of extraglandular disease expression, rather than as pathways dedicated to any single organ [17,20,22,29]. This distinction matters clinically, since cytokine profiles may help define disease endotypes but do not, by themselves, account for the specific organ phenotype expressed in an individual patient [22,29,38].

A recent Mendelian randomization study linked several inflammatory cytokines, including CXCL10, IL-4, IL-7, monocyte chemoattractant protein-2 (MCP-2), and tumor necrosis factor receptor superfamily member 9 (TNFRSF9), may be implicated in susceptibility to SjD [41]. However, these findings remain exploratory and should not be interpreted as evidence that these molecules constitute core pathogenic cytokine axes. They are therefore better regarded as emerging cytokine candidates or potential biomarkers requiring validation in tissue-based and longitudinal studies.

## 4. Cytokines Associated with Constitutional Symptoms

Patient-reported symptoms—including fatigue, pain, and dryness—are the major contributors to impaired quality of life in SjD [30,42]. These symptoms are not fully accounted for by the classical inflammatory cytokine network alone, as symptom burden frequently dissociates from objective disease measures, including systemic disease activity, glandular dysfunction, and inflammatory protein profiles [30,42,43]. Thus, a more nuanced framework is required, one that integrates both cytokine-driven and non-inflammatory mechanisms to comprehensively explain the symptom burden in SjD [43].

This discrepancy complicates the interpretation of proteomic findings associated with patient-reported symptoms. Patients with SjD and those with non-SjD sicca exhibited similar ESSPRI symptom burdens, but distinct differences were observed in their symptom-associated proteomic signatures [43]. Furthermore, no clear relationship was observed between inflammatory proteins and symptom severity in patients with SjD. These findings suggest that constitutional symptoms in SjD, including fatigue, pain, and dryness, do not simply represent an extension of systemic inflammatory activity [43,44,45]. A notable dissociation exists between objective disease activity, as measured by ESSDAI, and patient-reported symptom burden, as captured by ESSPRI; conversely, relatively low symptom burden can be observed even in the setting of overt inflammatory activity [46,47]. Therefore, constitutional symptoms in SjD are better understood as the product of interplay between inflammatory pathways and non-inflammatory symptom mechanisms, rather than being attributable to cytokine-driven inflammation alone.

Metabolomic and lipidomic data add another piece to this symptom-inflammation puzzle [48,49,50,51]. One labial salivary gland metabolomic study identified an immuno-lipidomic signature—kynurenine (KYN) plus several phospholipids—that separated early pSjD from non-pSjD sicca, hinting that metabolic shifts in glandular tissue can precede full-blown structural damage [50]. Another study reported lipid profiling of saliva, tears, and minor salivary glands has likewise turned up altered lipid composition in SjD patients, pointing to lipid-related mediators as players in glandular dysfunction and local immune signaling [49]. Saliva metabolomic work has flagged altered amino acid and lipid pathways as candidate biomarkers separating SjD from controls [48]. Tryptophan metabolites feed into immune regulation via the KYN pathway, and KYN metabolism itself appears disrupted in pSjD [33,51,52,53]. Taken together, elevated pro-inflammatory lipid mediators, shifted amino acid metabolites, and higher tryptophan metabolite levels may explain why dryness, fatigue, and pain do not always track with objective disease activity [33,51,52,53]. But these metabolic signatures are still exploratory and shouldn’t be read as established causal mechanisms [48,49,50,51].

However, cytokines are not entirely unrelated to constitutional symptoms. Certain pro-inflammatory cytokines, particularly IFN-γ, activate the KYN pathway via indoleamine 2,3-dioxygenase (IDO1), which may contribute to pain sensitization, dysesthesia, depressive symptoms, and fatigue through modulation of serotonergic and glutamatergic neurotransmission [52,53]. Supporting this, recent studies have reported that the KYN/tryptophan ratio correlates with fatigue severity in patients with SjD, positioning it as a candidate neuroimmune–metabolic biomarker [33]. Nevertheless, as the relationships among IFN-γ, IDO1, and KYN metabolites have not been consistently established in SjD, this pathway should be regarded as a candidate mechanism rather than a confirmed causative link [33,51,52,53].

Adrenomedullin (ADM), soluble CD40, and spondin-2 (SPON2) are symptom-associated proteins proposed by Pucino et al. as supplementary mediators of constitutional symptoms [43]. A proteomic study revealed that these proteins correlated with symptom variability in sicca, with ADM showing a particularly strong correlation with ESSPRI [43]. Notably, however, these correlations were more prominent in non-SjD sicca than in SjD itself, suggesting that interpreting these molecules as SjD-specific cytokine markers would be an overinterpretation [43]. Metabolomic work in parallel has picked up shifts in tryptophan-KYN metabolism and lipid-related signatures in SjD, raising the possibility that symptom burden owes something to neuroimmune-metabolic or non-inflammatory mechanisms beyond classical cytokine activity [33,50,51]. Taken as a whole, these findings point to classical inflammatory cytokines falling short of fully explaining constitutional symptoms like dryness, pain, and fatigue, with non-inflammatory, proteomic, or metabolic drivers possibly playing a larger role in symptom burden for a subset of patients [33,43,50,51]. Table 1 summarizes the cytokine networks tied to glandular inflammation, systemic immune activation, and constitutional symptoms.

## 5. Cytokine-Targeted Therapeutic Implications

The cytokine network underlying SS provides a strong biological rationale for the development of targeted therapies; however, clinical trial outcomes have been inconsistent, highlighting the complexity of translating pathogenic mechanisms into effective treatments [11,12,13,14,15,16,54]. These inconsistent outcomes likely reflect the marked heterogeneity of SjD, wherein glandular inflammation, systemic immune activation, B cell hyperactivity, and constitutional symptoms contribute to disease expression to varying degrees among individual patients [22,30,38,42,43]. Accordingly, the efficacy of cytokine-targeted therapies should be assessed within biologically defined disease subgroups—such as the IFN-high, B cell-dominant, and symptom-dominant phenotypes—rather than assuming a uniform therapeutic response across the entire SjD population [22,29,35,38,43].

Clinical heterogeneity also shows up in the split between pSjD and associated SjD [2,3]. Most mechanistic studies and therapeutic trials have zeroed in on pSjD, while associated SjD may be shaped by the cytokine milieu and treatment context of the accompanying SARD [2,11]. Going forward, cytokine-targeted strategies should either limit enrollment to a clearly defined SjD population or build in prespecified subgroup analyses by primary versus associated disease status [3,11].

For associated SjD, decisions on cytokine-targeted therapy should not be dictated by sicca or SjD-specific features alone but should account for the underlying SARD, its dominant organ involvement, and the therapies already in use for that disease [2,3,11]. In SjD associated with rheumatoid arthritis, systemic lupus erythematosus, or systemic sclerosis, for instance, treatment choice may be shaped by the cytokine pathways and approved agents relevant to the coexisting disease [55,56,57]. Associated SjD should therefore be regarded as a clinically distinct context for therapeutic stratification rather than managed as a direct extension of pSjD [2,3].

Failure to achieve clinical benefit in SS does not necessarily indicate that the selected therapeutic target is inappropriate. Rather, it may stem from the fact that ESSDAI, ESSPRI, glandular function, and serologic activity each capture distinct disease domains, making it inherently difficult to evaluate the efficacy of cytokine-targeted therapy through a single outcome measure [30,42,54]. For instance, in patients with overt B cell hyperactivity, serologic markers and systemic disease activity may improve, whereas fatigue and dryness may not respond to the same degree [11,16,30,42,43].

The IFN–JAK/STAT axis represents one of the most well-defined translational targets in SjD [22,35]. Type I IFN signature and JAK–STAT activation have been observed in both salivary gland tissue and PBMCs, with some evidence linking these findings to glandular inflammation and systemic immune activation [22,35,36]. JAK inhibition has been proposed as a therapeutic strategy because it suppresses IFN-driven immune activation [15,35]. However, as the IFN signature is not uniformly present across all SjD patients, future studies will require a biomarker strategy to prospectively identify and enrich for the IFN-high phenotype [22,35,38].

Drug repurposing has also surfaced as a complementary route for finding therapeutic candidates in SjD [58,59]. Conventional drug development in SjD has been held back by clinical heterogeneity, inconsistent trial endpoints, and the lack of validated molecular stratification [11,54]. Transcriptomic and computational repurposing approaches can help prioritize existing drugs that hit relevant immune pathways [58,59]. One transcriptomic drug-repurposing study flagged 11 candidate drugs for pSjD, with histone deacetylase and phosphoinositide 3-kinase inhibitors standing out as the most significantly associated drug classes [58]. Another separate integrative transcriptomic drug-repositioning analysis spanning systemic autoimmune diseases, SjD included, turned up six small-molecule candidates and pointed to the PI3K/AKT pathway as a recurring targetable node [59]. These results are best read as hypothesis-generating rather than proof of clinical efficacy, and the candidate drugs still need validation in experimental models and biomarker-enriched clinical trials [58,59].

The BAFF/APRIL pathway has emerged as a principal therapeutic target for the B cell-dominant phenotype of SS because of its critical role in promoting B cell survival, maintaining plasma cells, and sustaining autoantibody production [17,20,28]. Ianalumab targets the BAFF receptor and showed clinical efficacy in a phase 2b trial in patients with pSjD [16]. Telitacicept targets BAFF/APRIL and has shown efficacy and safety in a randomized phase 2 trial in pSjD [13]. This approach suggests that B cell-targeted therapy is not intended to address all manifestations of SjD but rather holds greater promise in the subgroup characterized by overt B cell hyperactivity [13,16,17,20].

T cell-related cytokines, including IL-6, IL-17, and IL-21, are involved in immune amplification in SjD; however, none of these pathways has yet been established as a confirmed therapeutic target [21,26,29,32]. Notably, IL-6 receptor inhibition has failed to demonstrate clear clinical efficacy in SjD despite its pathophysiological rationale, highlighting the potential limitations of single cytokine blockade in this disease [14]. Although glandular inflammation, systemic immune activation, B cell hyperactivity, and constitutional symptoms are overlapping and intertwined, they are not governed by a single shared mechanism [7,17,22,29,43]. Therefore, future clinical trials will require patient stratification integrating cytokine profiles, tissue transcriptomics, serologic B cell activity, ESSDAI, and ESSPRI to better match therapeutic targets to disease phenotypes [35,54].

Future clinical trials should begin by clearly defining the disease domain of interest before patient enrollment to ensure that therapeutic strategies are evaluated within the appropriate biological context [54]. Patients with an IFN-high phenotype may be more appropriate candidates for IFN–JAK/STAT-targeted therapy, whereas those with hypergammaglobulinemia, hypocomplementemia, cryoglobulinemia, or ectopic lymphoid organization may be better suited for B cell-directed strategies [22,35]. In contrast, patients with a symptom-dominant phenotype and low inflammatory activity may require distinct outcome measures and adjunctive non-immunologic approaches rather than conventional cytokine-targeted therapy [11,30,33,42,43,52,53,54]. The major cytokine-targeted approaches, their therapeutic rationale, potentially relevant phenotypes, and current limitations are summarized in Table 2.

**Figure 2:**
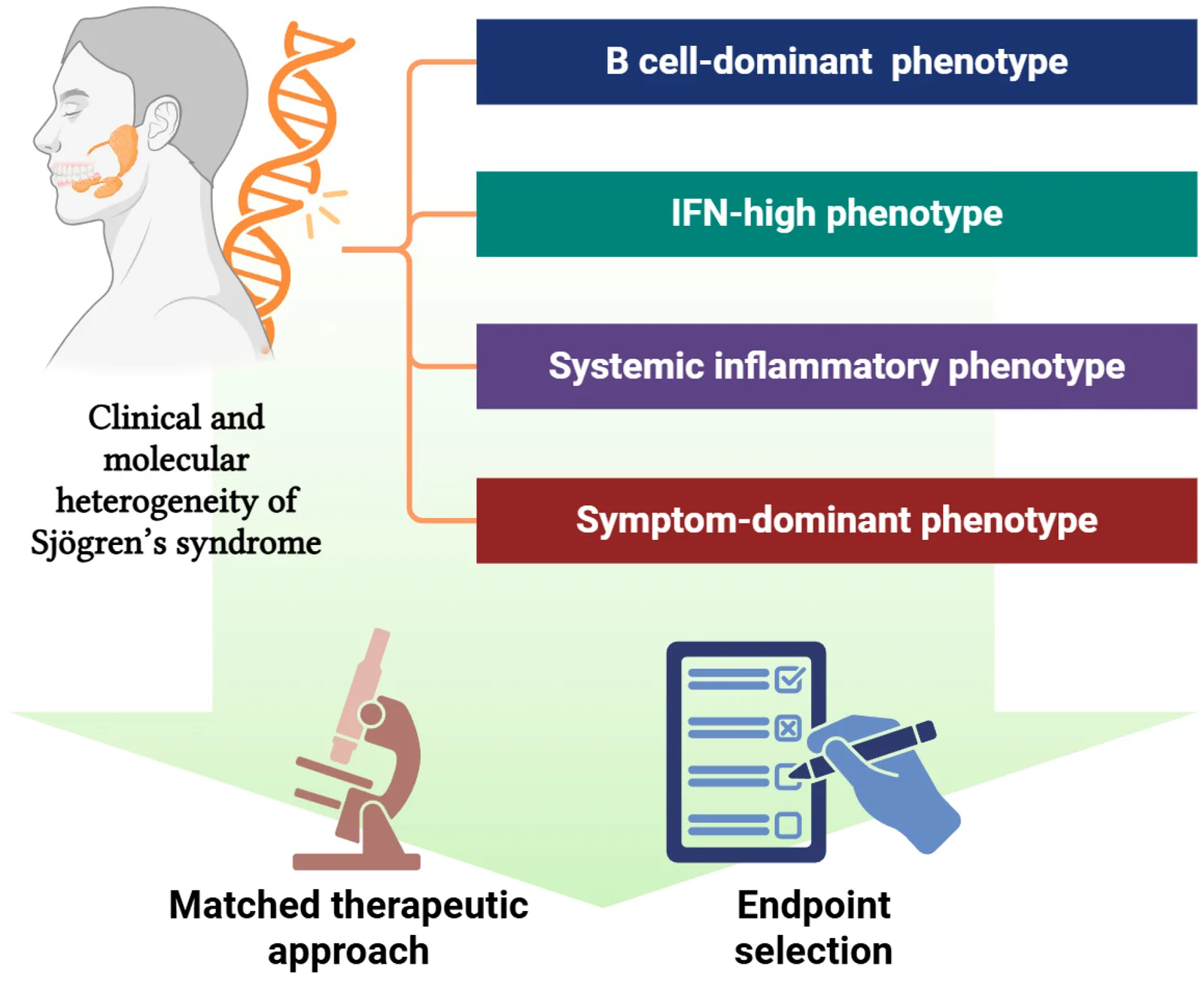
Cytokine profile-based therapeutic stratification in Sjögren’s syndrome. SjD is a heterogeneous disease in which glandular inflammation, systemic immune activation, B cell hyperactivity, and constitutional symptoms contribute in different proportions across individual patients. Patients with an IFN-high phenotype may be candidates for IFN–JAK/STAT-targeted approaches, whereas those with B cell-dominant features may be more suitable for BAFF/APRIL or B cell-targeted strategies. Patients with a systemic inflammatory phenotype may require outcome assessment focused on ESSDAI and organ-specific manifestations. In contrast, patients with symptom-dominant disease and low inflammatory activity may require symptom-oriented outcome measures and adjunctive non-immunologic approaches. This model highlights the need to match therapeutic targets and clinical trial endpoints to the dominant disease domain. Abbreviations: APRIL, a proliferation-inducing ligand; BAFF, B cell-activating factor; ESSDAI, EULAR Sjögren’s Syndrome Disease Activity Index; EULAR, European Alliance of Associations for Rheumatology; IFN, interferon; JAK, Janus kinase; SjD, Sjögren’s disease; STAT, signal transducer and activator of transcription.

Another important issue in cytokine-targeted therapy is the selection of appropriate outcome measures. A treatment that reduces systemic inflammation or serologic B cell activity may not lead to parallel improvement in fatigue, pain, or dryness. Conversely, improvement in patient-reported symptoms may occur without clear changes in systemic inflammatory markers. This discordance may partly explain why several biologically rational therapeutic strategies have produced only modest or inconsistent clinical benefits in trials involving patients with SjD. Future studies should therefore define the target domain before enrollment and select outcome measures accordingly, distinguishing systemic activity, glandular function, serologic activity, and patient-reported symptom burden. A proposed framework linking dominant cytokine profiles, potentially relevant therapeutic approaches, and outcome selection is shown in Figure 2 [11,30,42].

## 6. Conclusions

The cytokine landscape of SjD is highly complex and cannot be adequately explained by a single pathogenic pathway [7,17,20,22,23,29,35,43]. Glandular inflammation arises from the convergence of SGEC activation, IFN signaling, Th17-related cytokines, BAFF/APRIL, and chemokine-mediated immune trafficking [7,17,19,20,21,22,23,28,32,34,35]. Systemic immune activation is primarily driven by the coordinated actions of the BAFF/APRIL pathway, the type I IFN signature, and IL-21-mediated Tfh cell–B cell interactions, which collectively promote B cell hyperactivity and autoantibody production [17,20,22,28,29]. In contrast, constitutional symptoms, including fatigue, pain, and dryness, do not consistently correlate with inflammatory cytokine activity and must be understood in the context of neuroimmune–metabolic pathways and symptom-associated proteomic signatures [33,43,48,49,50,51,52,53].

Future research and clinical trials should adopt an integrated, phenotype-driven approach that interprets cytokine profiles within specific clinical domains to facilitate more precise biomarker selection, therapeutic targeting, and outcome assessment [17,22,29,35,36,38,43]. Such a domain-based approach may help refine biomarker selection, therapeutic targeting, and outcome assessment in future SjD trials [11,12,13,14,15,30,42,43,54].

## Figures and Tables

**Table 1:** Cytokine networks according to clinical domains in Sjögren’s syndrome.

Clinical Domain	Cytokines or Molecules	Cellular or Molecular Context	Clinical Relevance
Glandular inflammation	IFN-α IFN-γ IL-6 IL-17 IL-22 CXCL10 CXCL12 CXCL13 BAFF	SGEC activation pDC activation Th1/Th17 response B cell recruitment chemokine-mediated trafficking	Lymphocytic infiltration Focus score Epithelial dysfunction Salivary/lacrimal gland dysfunction
Systemic immune activation	Type I IFN signature BAFF APRIL IL-21 IL-6 IL-17	B cell hyperactivity Plasmablast/plasma cell expansion Tfh–B cell interaction Systemic IFN activation	Anti-Ro/La positivity Hypergammaglobulinemia Hypocomplementemia Cryoglobulinemia Lymphoma-prone phenotype
Constitutional symptoms	IFN-γ–IDO–KYN pathway ADM soluble CD40 SPON2 Lipid-related mediators TRP and other amino acid metabolites	Neuroimmune–metabolic interaction Symptom-associated proteomic signature Non-inflammatory symptom mechanisms Metabolomic/lipidomic signature	Fatigue Pain Dryness ESSPRI Symptom–inflammation dissociation

Abbreviations: ADM, adrenomedullin; APRIL, a proliferation-inducing ligand; BAFF, B cell-activating factor; CD40, cluster of differentiation 40; CXCL, C-X-C motif chemokine ligand; ESSPRI, EULAR Sjögren’s Syndrome Patient Reported Index; EULAR, European Alliance of Associations for Rheumatology; IDO, indoleamine 2,3-dioxygenase; IFN, interferon; IL, interleukin; KYN, kynurenine; pDC, plasmacytoid dendritic cell; SGEC, salivary gland epithelial cell; SPON2, spondin-2; Th, T helper; Tfh, follicular helper T cell; TRP, tryptophan.

**Table 2:** Cytokine-targeted therapeutic implications in Sjögren’s syndrome.

Target Axis	Therapeutic Rationale	Potentially Relevant Phenotype	Current Limitation
IFN–JAK/STAT axis	Suppression of IFN-driven immune activation and ISG expression	IFN-high phenotype, glandular/systemic inflammatory phenotype	IFN signature is heterogeneous and requires biomarker-based enrichment
BAFF/APRIL axis	Inhibition of B cell survival, plasma-cell maintenance, and autoantibody production	B cell-dominant phenotype, anti-Ro/La positivity, hypergammaglobulinemia, hypocomplementemia, cryoglobulinemia	B cell targeting may not improve constitutional symptoms proportionally
IL-6 pathway	Modulation of Th17 differentiation and inflammatory amplification	Systemic inflammatory phenotype	Clinical efficacy has not been clearly demonstrated
IL-17/Th17-related pathway	Reduction in Th17-mediated epithelial and inflammatory responses	Glandular inflammatory phenotype; exploratory	Not established as a validated therapeutic target in SjD
Symptom-associated pathways	Targeting neuroimmune–metabolic mechanisms rather than classical inflammation	Symptom-dominant phenotype with fatigue, pain, dryness	Mechanisms remain exploratory; endpoints should include ESSPRI, fatigue, pain, and dryness measures
Drug repurposing approaches	Transcriptomic or computational prioritization of existing drugs targeting immune or signaling pathways	Pathway-enriched or biomarker-defined subgroups	Hypothesis-generating; requires experimental and clinical validation

Abbreviations: APRIL, a proliferation-inducing ligand; BAFF, B cell-activating factor; ESSPRI, EULAR Sjögren’s Syndrome Patient Reported Index; EULAR, European Alliance of Associations for Rheumatology; IFN, interferon; IL, interleukin; ISG, interferon-stimulated gene; JAK, Janus kinase; STAT, signal transducer and activator of transcription; Th, T helper.

## Data Availability

No new data were created or analyzed in this study. Data sharing is not applicable to this article.

## Abbreviations

The following abbreviations are used in this manuscript:

ACR	American College of Rheumatology
ADM	Adrenomedullin
APC	Antigen-presenting cell
APRIL	A proliferation-inducing ligand
BAFF	B cell-activating factor
BDCA-2	Blood dendritic cell antigen 2
BLK	B lymphocyte kinase
Breg	Regulatory B cell
CD	Cluster of differentiation
CMV	Cytomegalovirus
CRP	C-reactive protein
CXCL	C-X-C motif chemokine ligand
CXCR5	C-X-C motif chemokine receptor 5
DAMP	Damage-associated molecular pattern
DC	dendritic cell
DNA	deoxyribonucleic acid
EBV	Epstein–Barr virus
ESR	erythrocyte sedimentation rate
ESSDAI	EULAR Sjögren’s Syndrome Disease Activity Index
ESSPRI	EULAR Sjögren’s Syndrome Patient Reported Index
EULAR	European Alliance of Associations for Rheumatology
HDAC	Histone deacetylase
HLA-DQB1	Human leukocyte antigen DQB1
HPLC/MS	High-performance liquid chromatography/mass spectrometry
ICAM-1	Intercellular adhesion molecule 1
IDO	Indoleamine 2,3-dioxygenase
IDO1	Indoleamine 2,3-dioxygenase 1
IFN	Interferon
IgG	Immunoglobulin G
IgM	Immunoglobulin M
IL	Interleukin
IL12A	Interleukin-12 subunit alpha
IRF5	Interferon regulatory factor 5
ISG	Interferon-stimulated gene
JAK	Janus kinase
KYN	Kynurenine
LysoPC	Lysophosphatidylcholine
MCP-2	Monocyte chemoattractant protein-2
MHC	Major histocompatibility complex
MMP-9	Matrix metalloproteinase-9
NK	Natural killer
PBMC	Peripheral blood mononuclear cell
PC	Phosphatidylcholine
pDC	Plasmacytoid dendritic cell
PI3K	Phosphoinositide 3-kinase
pSjD	Primary Sjögren’s disease
RNA	Ribonucleic acid
SARD	Systemic autoimmune rheumatic disease
SGEC	Salivary gland epithelial cell
SPON2	Spondin-2
SjD	Sjögren’s disease
STAT	Signal transducer and activator of transcription
STAT4	Signal transducer and activator of transcription 4
Th	T helper
Tfh	Follicular helper T cell
Tfr	Follicular regulatory T cell
Th1	T helper 1
Th2	T helper 2
Th17	T helper 17
TLR	Toll-like receptor
TNF-α	Tumor necrosis factor-α
TNFAIP3	Tumor necrosis factor alpha-induced protein 3
TNFRSF9	Tumor necrosis factor receptor superfamily member 9
TNIP1	TNFAIP3-interacting protein 1
Treg	Regulatory T cell
TRP	Tryptophan
VCAM-1	Vascular cell adhesion molecule 1

## References

[1] Shiboski C.H., Shiboski S.C., Seror R., Criswell L.A., Labetoulle M., Lietman T.M., Rasmussen A., Scofield H., Vitali C., Bowman S.J. (2017). 2016 American College of Rheumatology/European League Against Rheumatism Classification Criteria for Primary Sjögren’s Syndrome: A Consensus and Data-Driven Methodology Involving Three International Patient Cohorts. Arthritis Rheumatol..

[2] Negrini S., Emmi G., Greco M., Borro M., Sardanelli F., Murdaca G., Indiveri F., Puppo F. (2022). Sjögren’s syndrome: A systemic autoimmune disease. Clin. Exp. Med..

[3] Ramos-Casals M., Baer A.N., Brito-Zerón M.D.P., Hammitt K.M., Bouillot C., Retamozo S., Mackey A., Yarowsky D., Turner B., Blanck J. (2025). 2023 International Rome consensus for the nomenclature of Sjögren disease. Nat. Rev. Rheumatol..

[4] Thorlacius G.E., Björk A., Wahren-Herlenius M. (2023). Genetics and epigenetics of primary Sjögren syndrome: Implications for future therapies. Nat. Rev. Rheumatol..

[5] Lessard C.J., Li H., Adrianto I., Ice J.A., Rasmussen A., Grundahl K.M., Kelly J.A., Dozmorov M.G., Miceli-Richard C., Bowman S. (2013). Variants at multiple loci implicated in both innate and adaptive immune responses are associated with Sjögren’s syndrome. Nat. Genet..

[6] Maslinska M., Kostyra-Grabczak K. (2022). The role of virus infections in Sjögren’s syndrome. Front. Immunol..

[7] Verstappen G.M., Pringle S., Bootsma H., Kroese F.G.M. (2021). Epithelial-immune cell interplay in primary Sjögren syndrome salivary gland pathogenesis. Nat. Rev. Rheumatol..

[8] Dong Y., Wang T., Wu H. (2024). The role of cytokines from salivary gland epithelial cells in the immunopathology of Sjögren’s syndrome. Front. Immunol..

[9] Shimizu T., Nakamura H., Takatani A., Umeda M., Horai Y., Kurushima S., Michitsuji T., Nakashima Y., Kawakami A. (2019). Activation of Toll-like receptor 7 signaling in labial salivary glands of primary Sjögren’s syndrome patients. Clin. Exp. Immunol..

[10] Spachidou M.P., Bourazopoulou E., Maratheftis C.I., Kapsogeorgou E.K., Moutsopoulos H.M., Tzioufas A.G., Manoussakis M.N. (2007). Expression of functional Toll-like receptors by salivary gland epithelial cells: Increased mRNA expression in cells derived from patients with primary Sjögren’s syndrome. Clin. Exp. Immunol..

[11] Ramos-Casals M., Brito-Zerón P., Bombardieri S., Bootsma H., De Vita S., Dörner T., Fisher B.A., Gottenberg J.E., Hernandez-Molina G., Kocher A. (2020). EULAR recommendations for the management of Sjögren’s syndrome with topical and systemic therapies. Ann. Rheum. Dis..

[12] St Clair E.W., Baer A.N., Ng W.F., Noaiseh G., Baldini C., Tarrant T.K., Papas A., Devauchelle-Pensec V., Wang L., Xu W. (2024). CD40 ligand antagonist dazodalibep in Sjögren’s disease: A randomized, double-blinded, placebo-controlled, phase 2 trial. Nat. Med..

[13] Xu D., Fang J., Zhang S., Huang C., Huang C., Qin L., Li X., Chen M., Liu X., Liu Y. (2024). Efficacy and safety of telitacicept in primary Sjögren’s syndrome: A randomized, double-blind, placebo-controlled, phase 2 trial. Rheumatology.

[14] Felten R., Devauchelle-Pensec V., Seror R., Duffau P., Saadoun D., Hachulla E., Pierre Yves H., Salliot C., Perdriger A., Morel J. (2021). Interleukin 6 receptor inhibition in primary Sjögren syndrome: A multicentre double-blind randomised placebo-controlled trial. Ann. Rheum. Dis..

[15] Bai W., Yang F., Xu H., Wei W., Li H., Zhang L., Zhao Y., Shi X., Zhang Y., Zeng X. (2023). A multi-center, open-label, randomized study to explore efficacy and safety of baricitinib in active primary Sjogren’s syndrome patients. Trials.

[16] Bowman S.J., Fox R., Dörner T., Mariette X., Papas A., Grader-Beck T., Fisher B.A., Barcelos F., De Vita S., Schulze-Koops H. (2022). Safety and efficacy of subcutaneous ianalumab (VAY736) in patients with primary Sjögren’s syndrome: A randomised, double-blind, placebo-controlled, phase 2b dose-finding trial. Lancet.

[17] Daridon C., Devauchelle V., Hutin P., Le Berre R., Martins-Carvalho C., Bendaoud B., Dueymes M., Saraux A., Youinou P., Pers J.O. (2007). Aberrant expression of BAFF by B lymphocytes infiltrating the salivary glands of patients with primary Sjögren’s syndrome. Arthritis Rheum..

[18] Ittah M., Miceli-Richard C., Eric Gottenberg J., Lavie F., Lazure T., Ba N., Sellam J., Lepajolec C., Mariette X. (2006). B cell-activating factor of the tumor necrosis factor family (BAFF) is expressed under stimulation by interferon in salivary gland epithelial cells in primary Sjögren’s syndrome. Arthritis Res. Ther..

[19] Blokland S.L.M., Flessa C.M., van Roon J.A.G., Mavragani C.P. (2021). Emerging roles for chemokines and cytokines as orchestrators of immunopathology in Sjögren’s syndrome. Rheumatology.

[20] Rivière E., Pascaud J., Tchitchek N., Boudaoud S., Paoletti A., Ly B., Dupré A., Chen H., Thai A., Allaire N. (2020). Salivary gland epithelial cells from patients with Sjögren’s syndrome induce B-lymphocyte survival and activation. Ann. Rheum. Dis..

[21] Loureiro-Amigo J., Franco-Jarava C., Perurena-Prieto J., Palacio C., Martínez-Valle F., Soláns-Laqué R. (2021). Serum CXCL13, BAFF, IL-21 and IL-22 levels are related to disease activity and lymphocyte profile in primary Sjögren’s syndrome. Clin. Exp. Rheumatol..

[22] Bodewes I.L.A., Al-Ali S., van Helden-Meeuwsen C.G., Maria N.I., Tarn J., Lendrem D.W., Schreurs M.W.J., Steenwijk E.C., van Daele P.L.A., Both T. (2018). Systemic interferon type I and type II signatures in primary Sjögren’s syndrome reveal differences in biological disease activity. Rheumatology.

[23] Alunno A., Carubbi F., Bistoni O., Caterbi S., Bartoloni E., Mirabelli G., Cannarile F., Cipriani P., Giacomelli R., Gerli R. (2015). T Regulatory and T Helper 17 Cells in Primary Sjögren’s Syndrome: Facts and Perspectives. Mediat. Inflamm..

[24] Xin X., Wang Q., Qing J., Song W., Gui Y., Li X., Li Y. (2022). Th17 cells in primary Sjögren’s syndrome negatively correlate with increased *Roseburia* and *Coprococcus*. Front. Immunol..

[25] Cano-Ortiz A., Laborda-Illanes A., Plaza-Andrades I., Membrillo Del Pozo A., Villarrubia Cuadrado A., Rodríguez Calvo de Mora M., Leiva-Gea I., Sanchez-Alcoholado L., Queipo-Ortuño M.I. (2020). Connection between the Gut Microbiome, Systemic Inflammation, Gut Permeability and FOXP3 Expression in Patients with Primary Sjögren’s Syndrome. Int. J. Mol. Sci..

[26] Choi J., Crotty S., Choi Y.S. (2024). Cytokines in Follicular Helper T Cell Biology in Physiologic and Pathologic Conditions. Immune Netw..

[27] Ewert P., Aguilera S., Alliende C., Kwon Y.J., Albornoz A., Molina C., Urzúa U., Quest A.F., Olea N., Pérez P. (2010). Disruption of tight junction structure in salivary glands from Sjögren’s syndrome patients is linked to proinflammatory cytokine exposure. Arthritis Rheum..

[28] Nocturne G., Mariette X. (2018). B cells in the pathogenesis of primary Sjögren syndrome. Nat. Rev. Rheumatol..

[29] Pontarini E., Murray-Brown W.J., Croia C., Lucchesi D., Conway J., Rivellese F., Fossati-Jimack L., Astorri E., Prediletto E., Corsiero E. (2020). Unique expansion of IL-21+ Tfh and Tph cells under control of ICOS identifies Sjögren’s syndrome with ectopic germinal centres and MALT lymphoma. Ann. Rheum. Dis..

[30] Seror R., Ravaud P., Mariette X., Bootsma H., Theander E., Hansen A., Ramos-Casals M., Dörner T., Bombardieri S., Hachulla E. (2011). EULAR Sjogren’s Syndrome Patient Reported Index (ESSPRI): Development of a consensus patient index for primary Sjogren’s syndrome. Ann. Rheum. Dis..

[31] Zhang L.W., Cong X., Zhang Y., Wei T., Su Y.C., Serrão A.C., Brito A.R., Yu G.Y., Hua H., Wu L.L. (2016). Interleukin-17 Impairs Salivary Tight Junction Integrity in Sjögren’s Syndrome. J. Dent. Res..

[32] Alunno A., Bistoni O., Bartoloni E., Caterbi S., Bigerna B., Tabarrini A., Mannucci R., Falini B., Gerli R. (2013). IL-17-producing CD4-CD8- T cells are expanded in the peripheral blood, infiltrate salivary glands and are resistant to corticosteroids in patients with primary Sjogren’s syndrome. Ann. Rheum. Dis..

[33] Park Y., Lee J.J., Koh J.H., Kim M.J., Park S.H., Kwok S.K. (2023). Kynurenine pathway can be a potential biomarker of fatigue in primary Sjögren’s syndrome. Clin. Exp. Rheumatol..

[34] Verstappen G.M., Corneth O.B.J., Bootsma H., Kroese F.G.M. (2018). Th17 cells in primary Sjögren’s syndrome: Pathogenicity and plasticity. J. Autoimmun..

[35] Gupta S., Yamada E., Nakamura H., Perez P., Pranzatelli T.J., Dominick K., Jang S.I., Abed M., Martin D., Burbelo P. (2024). Inhibition of JAK-STAT pathway corrects salivary gland inflammation and interferon driven immune activation in Sjögren’s disease. Ann. Rheum. Dis..

[36] Verstappen G.M., Gao L., Pringle S., Haacke E.A., van der Vegt B., Liefers S.C., Patel V., Hu Y., Mukherjee S., Carman J. (2021). The Transcriptome of Paired Major and Minor Salivary Gland Tissue in Patients with Primary Sjögren’s Syndrome. Front. Immunol..

[37] Huang J., Tang J., Zhang C., Liu T., Deng Z., Liu L. (2024). Single-cell transcriptomic analysis uncovers heterogeneity in the labial gland microenvironment of primary Sjögren’s syndrome. J. Transl. Autoimmun..

[38] James J.A., Guthridge J.M., Chen H., Lu R., Bourn R.L., Bean K., Munroe M.E., Smith M., Chakravarty E., Baer A.N. (2020). Unique Sjögren’s syndrome patient subsets defined by molecular features. Rheumatology.

[39] Mandl T., Marsal J., Olsson P., Ohlsson B., Andréasson K. (2017). Severe intestinal dysbiosis is prevalent in primary Sjögren’s syndrome and is associated with systemic disease activity. Arthritis Res. Ther..

[40] Lin X., Rui K., Deng J., Tian J., Wang X., Wang S., Ko K.H., Jiao Z., Chan V.S., Lau C.S. (2015). Th17 cells play a critical role in the development of experimental Sjögren’s syndrome. Ann. Rheum. Dis..

[41] Shi W., Xu Y., Zhang A., Jia X., Liu S., Hu Z. (2024). Inflammatory cytokines and their potential role in Sjogren’s syndrome risk: Insights from a mendelian randomization study. Adv. Rheumatol..

[42] Seror R., Theander E., Brun J.G., Ramos-Casals M., Valim V., Dörner T., Bootsma H., Tzioufas A., Solans-Laqué R., Mandl T. (2015). Validation of EULAR primary Sjögren’s syndrome disease activity (ESSDAI) and patient indexes (ESSPRI). Ann. Rheum. Dis..

[43] Pucino V., Turner J.D., Nayar S., Kollert F., Rauz S., Richards A., Higham J., Poveda-Gallego A., Bowman S.J., Barone F. (2022). Sjögren’s and non-Sjögren’s sicca share a similar symptom burden but with a distinct symptom-associated proteomic signature. RMD Open.

[44] Howard Tripp N., Tarn J., Natasari A., Gillespie C., Mitchell S., Hackett K.L., Bowman S.J., Price E., Pease C.T., Emery P. (2016). Fatigue in primary Sjögren’s syndrome is associated with lower levels of proinflammatory cytokines. RMD Open.

[45] Koh J.H., Kwok S.K., Lee J., Son C.N., Kim J.M., Kim H.O., Park S.H., Sung Y.K., Choe J.Y., Lee S.S. (2017). Pain, xerostomia, and younger age are major determinants of fatigue in Korean patients with primary Sjögren’s syndrome: A cohort study. Scand. J. Rheumatol..

[46] Ture H.Y., Kim N.R., Nam E.J. (2023). EULAR Sjogren’s Syndrome Patient Reported Index (ESSPRI) and Other Patient-Reported Outcomes in the Assessment of Glandular Dysfunction in Primary Sjögren’s Syndrome. Life.

[47] Tarn J.R., Howard-Tripp N., Lendrem D.W., Mariette X., Saraux A., Devauchelle-Pensec V., Seror R., Skelton A.J., James K., McMeekin P. (2019). Symptom-based stratification of patients with primary Sjögren’s syndrome: Multi-dimensional characterisation of international observational cohorts and reanalyses of randomised clinical trials. Lancet Rheumatol..

[48] Li Z., Mu Y., Guo C., You X., Liu X., Li Q., Sun W. (2022). Analysis of the saliva metabolic signature in patients with primary Sjogren’s syndrome. PLoS ONE.

[49] Fineide F., Chen X., Bjellaas T., Vitelli V., Utheim T.P., Jensen J.L., Galtung H.K. (2021). Characterization of Lipids in Saliva, Tears and Minor Salivary Glands of Sjogren’s Syndrome Patients Using an HPLC/MS-Based Approach. Int. J. Mol. Sci..

[50] Urbanski G., Chabrun F., Delattre E., Lacout C., Davidson B., Blanchet O., Chao de la Barca J.M., Simard G., Lavigne C., Reynier P. (2023). An immuno-lipidomic signature revealed by metabolomic and machine-learning approaches in labial salivary gland to diagnose primary Sjogren’s syndrome. Front. Immunol..

[51] Eryavuz Onmaz D., Tezcan D., Abusoglu S., Sak F., Humeyra Yerlikaya F., Yilmaz S., Abusoglu G., Kazim Korez M., Unlu A. (2023). Impaired kynurenine metabolism in patients with primary Sjogren’s syndrome. Clin. Biochem..

[52] Maria N.I., van Helden-Meeuwsen C.G., Brkic Z., Paulissen S.M., Steenwijk E.C., Dalm V.A., van Daele P.L., Martin van Hagen P., Kroese F.G., van Roon J.A. (2016). Association of Increased Treg Cell Levels with Elevated Indoleamine 2,3-Dioxygenase Activity and an Imbalanced Kynurenine Pathway in Interferon-Positive Primary Sjögren’s Syndrome. Arthritis Rheumatol..

[53] Pertovaara M., Raitala A., Uusitalo H., Pukander J., Helin H., Oja S.S., Hurme M. (2005). Mechanisms dependent on tryptophan catabolism regulate immune responses in primary Sjögren’s syndrome. Clin. Exp. Immunol..

[54] Arends S., de Wolff L., van Nimwegen J.F., Verstappen G., Vehof J., Bombardieri M., Bowman S.J., Pontarini E., Baer A.N., Nys M. (2021). Composite of Relevant Endpoints for Sjögren’s Syndrome (CRESS): Development and validation of a novel outcome measure. Lancet Rheumatol..

[55] Smolen J.S., Landewé R.B.M., Bergstra S.A., Kerschbaumer A., Sepriano A., Aletaha D., Caporali R., Edwards C.J., Hyrich K.L., Pope J.E. (2023). EULAR recommendations for the management of rheumatoid arthritis with synthetic and biological disease-modifying antirheumatic drugs: 2022 update. Ann. Rheum. Dis..

[56] Del Galdo F., Lescoat A., Conaghan P.G., Bertoldo E., Čolić J., Santiago T., Suliman Y.A., Matucci-Cerinic M., Gabrielli A., Distler O. (2025). EULAR recommendations for the treatment of systemic sclerosis: 2023 update. Ann. Rheum. Dis..

[57] Fanouriakis A., Kostopoulou M., Andersen J., Aringer M., Arnaud L., Bae S.C., Boletis J., Bruce I.N., Cervera R., Doria A. (2024). EULAR recommendations for the management of systemic lupus erythematosus: 2023 update. Ann. Rheum. Dis..

[58] Felten R., Ye T., Schleiss C., Schwikowski B., Sibilia J., Monneaux F., Dumortier H., Jonsson R., Lessard C., Ng F. (2023). Identification of new candidate drugs for primary Sjogren’s syndrome using a drug repurposing transcriptomic approach. Rheumatology.

[59] Oktem E.K., Yazar M. (2022). Drug Repositioning Identifies Six Drug Candidates for Systemic Autoimmune Diseases by Integrative Analyses of Transcriptomes from Scleroderma, Systemic Lupus Erythematosus, and Sjogren’s Syndrome. OMICS.

